# Supplementary material for: The mechanistic and functional profile of the therapeutic anti-IgE antibody ligelizumab differs from omalizumab
Source: Nat Commun. 2020 Jan 8;11:165. doi: 10.1038/s41467-019-13815-w (PMC6949303; doi:10.1038/s41467-019-13815-w)
Supplement: Supplementary file 1 — Supplementary Information [file 41467_2019_13815_MOESM1_ESM.pdf]

Supplementary Information

The mechanistic and functional profile of the therapeutic anti-IgE antibody ligelizumab differs from omalizumab

Gasser et al.

Supplementary Figures

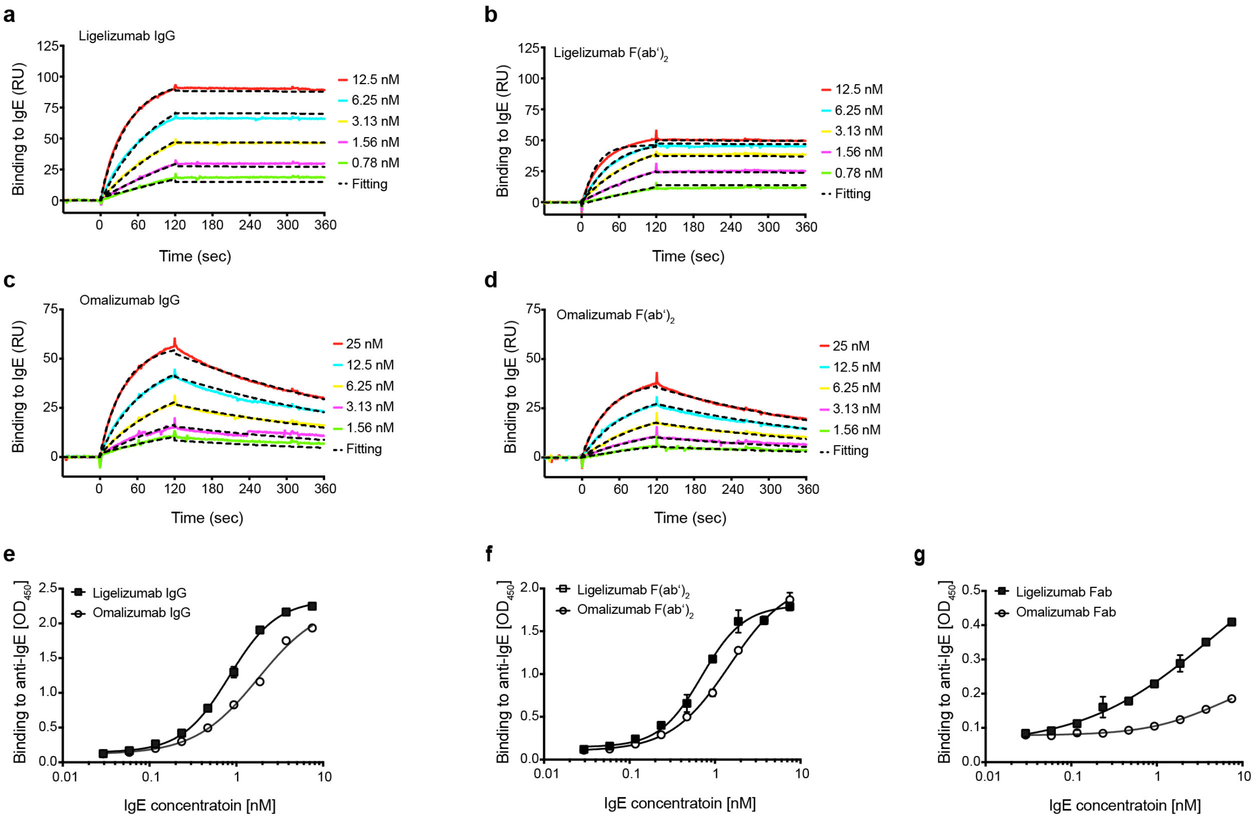

Supplementary Figure 1 | Binding kinetics of ligelizumab or omalizumab IgG and Fab on recombinant human IgE. (a,b) Dose- and time-dependent association and dissociation of ligelizumab (**a,b**) and omalizumab (**c,d**) IgG and $F(ab')_2$ fragments on Le27 captured human recombinant IgE-Sus11 was measured by SPR. Each color in the sensorgram refers to an individual measurement cycle for the indicated concentration of anti-IgE antibodies. Curves were fitted (black dashed lines) using a 1:1 langmuir binding model. (**e-g**) Binding of ligelizumab and omalizumab IgG (**e**) as well as $F(ab')_2$ (**f**) and Fab fragments (**g**) on immobilized recombinant human IgE-Sus11 was measured by ELISA. Binding curves were fitted using a non-linear regression model (data shown for technical duplicates as mean ± SEM). Source data are provided as Source Data file.

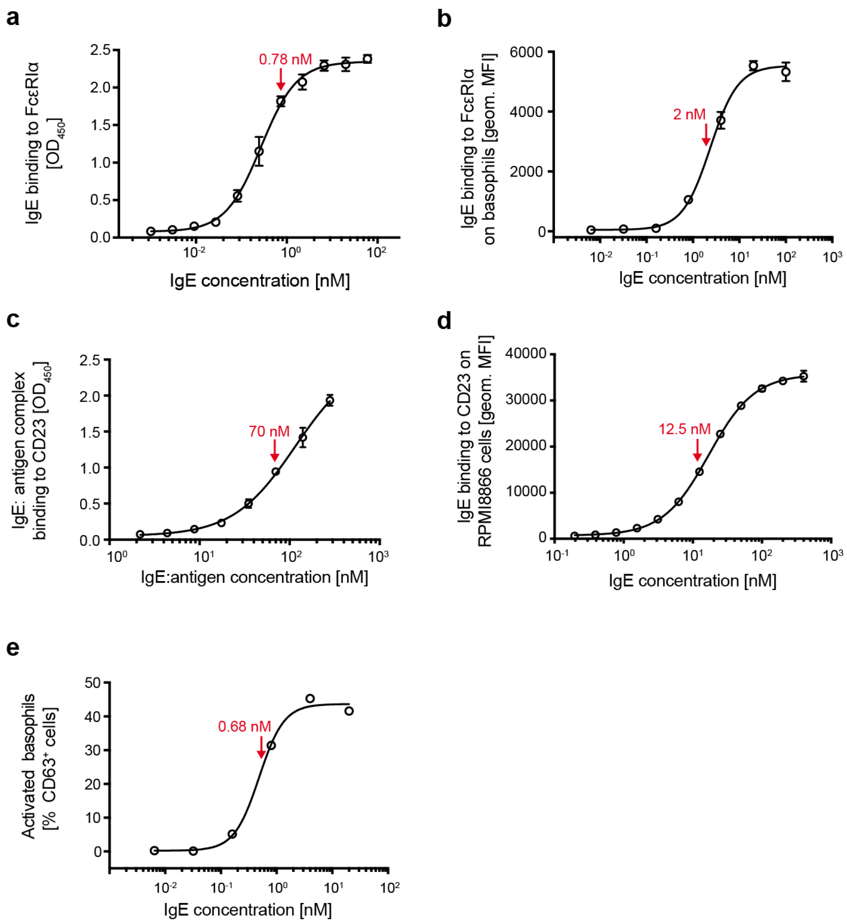

Supplementary Figure 2 | Titration of IgE on recombinant or cell surface expressed FcεRIα and CD23. (**a**) Dose-dependent binding of recombinant human IgE-Sus11 to immobilized recombinant FcεRIα was measured by ELISA. The red arrow indicates the IgE concentration used for subsequent inhibition of IgE binding to FcεRIα by ligelizumab IgG or omalizumab IgG. (**b)** Dose-dependent binding of recombinant human JW8-IgE on bi53_79 treated isolated primary human basophils were assessed by flow cytometry. The red arrow indicated the concentration of Sus11-IgE, which was used for subsequent inhibition of IgE binding to FcεRIα on isolated bi53_79 treated basophils by ligelizumab IgG or omalizumab IgG. (**c**) Dose-dependent binding of chimeric human IgE-JW8 complexed with NIP_7-BSA in a 1:1 ratio to immobilized human recombinant CD23 was measured by ELISA. The red arrow indicates the IgE:antigen complex concentration used for subsequent inhibition of IgE:antigen complex binding to CD23 by ligelizumab IgG or omalizumab IgG. (**d**) Dose-dependent binding of IgE to RPMI8866 cells was measured by flow cytometry. The red arrow indicates the concentration used for subsequent inhibition of IgE binding to RPMI8866 cells by ligelizumab IgG or omalizumab IgG. (**e**) IgE-dependent activation of JW8-IgE resensitized isolated $CD193^+$ primary human basophils stimulated with 100 ng/ml NIP_7-BSA. The red arrow indicates the concentration of IgE-JW8 which was used for the subsequent inhibition of NIP_7-BSA-mediated activation of isolated $CD193^+$ primary human basophils by ligelizumab IgG or omalizumab IgG. Binding curves for were fitted using a non-linear regression model (data shown for technical duplicates as mean ± SEM). Source data are provided as Source Data file.

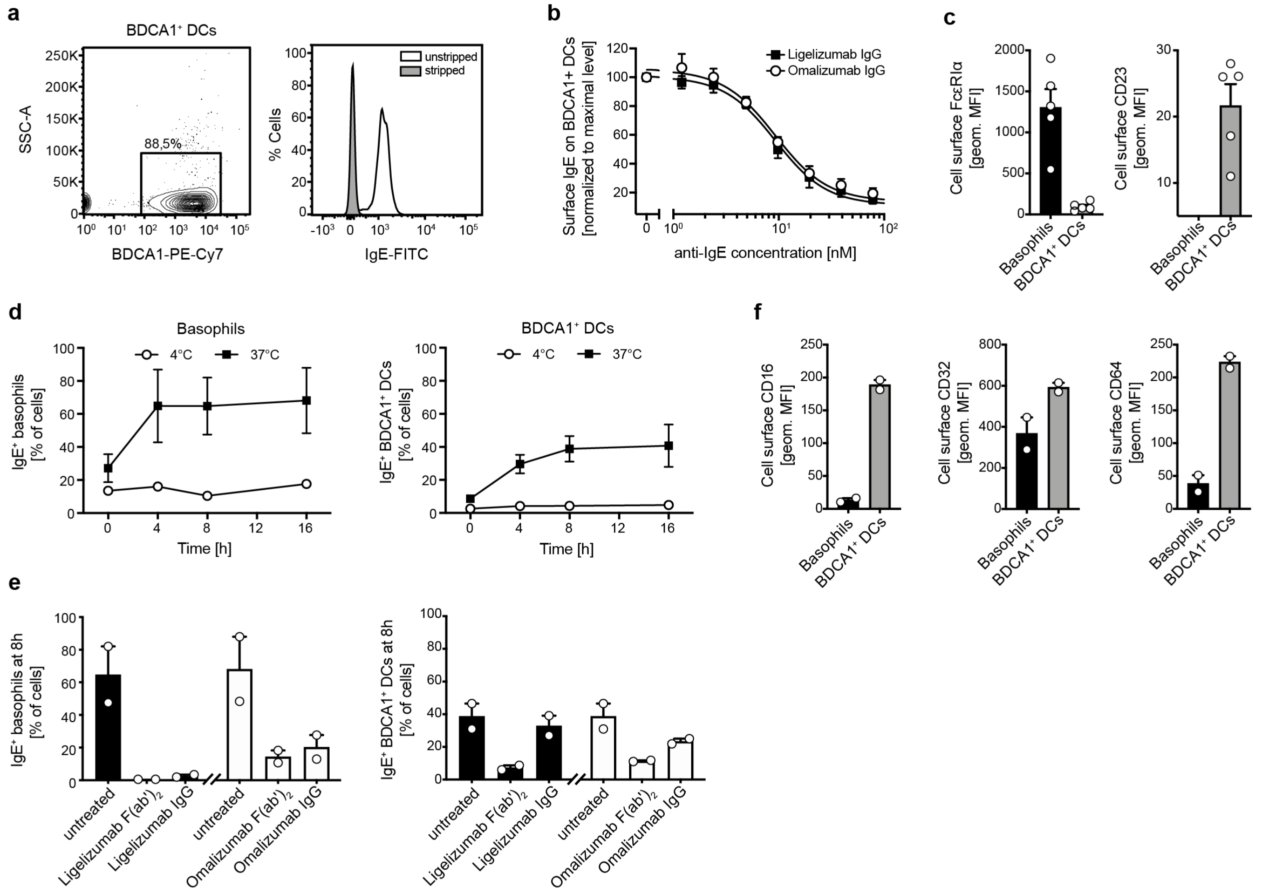

Supplementary Figure 3 | Inhibition of IgE binding and IgE:antigen complex internalization by ligelizumab or omalizumab. (a) Representative flow cytometry plot and histogram showing isolated BDCA1$^+$ DCs that were treated with disruptive DARPin® protein bi53_79 to remove surface bound IgE (stripped) or left untreated (unstripped). (**b**) Dose-dependent inhibition of IgE binding stripped BDCA1$^+$ DCs by ligelizumab IgG or omalizumab IgG. Binding curves were fitted using a non-linear regression model. Data are shown as mean ± SEM (*n*=4 donors). (**c**) Flow cytometric analysis of FcεRIα and CD23 expression on isolated basophils and BDCA1$^+$ DCs. Data are shown as mean ± SEM (*n*=5 donors). (**d**) Flow cytometric quantification of time-dependent IgE:antigen complex internalization with isolated basophils or BDCA1$^+$ DCs at 37°C or 4 °C. (**e**) Flow cytometric quantification of IgE:antigen complex internalization after 8 hours post pre-incubation of the complexes with ligelizumab IgG or F(ab')2 fragments and omalizumab IgG or F(ab')2 fragments with isolated basophils or BDCA1$^+$ DCs at 37°C. (**f**) Flow cytometric analysis of CD16, CD32 and CD64 IgG-receptor expression on isolated basophils and BDCA1$^+$ DCs. (**d-f**) Data are shown as mean ± SEM (*n*=2 donors). Source data are provided as Source Data file.

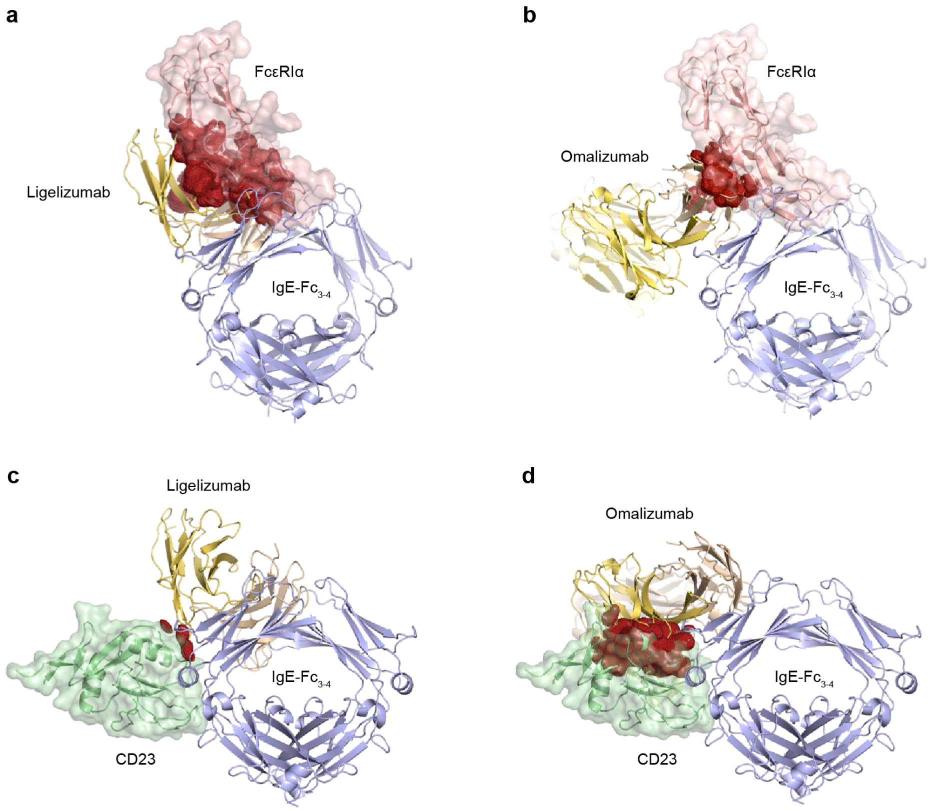

Atomic Overlap Volume (Å^3)	
	**FcεRIα**
**Ligelizumab**	4622
**Omalizumab**	1063

Atomic Overlap Volume (Å^3)	
	**CD23**
**Ligelizumab**	28
**Omalizumab**	2773

Supplementary Figure 4 | Comparison of the predicted steric overlap of ligelizumab and omalizumab complexes with FcεRIα and CD23. Superpositions of the IgE complexes with FcεRIα (**a, b**) or CD23 (**c, d**) and ligelizumab or omalizumab. The complexes were superimposed using the receptor-proximal Cε3 domains. Atomic overlap volumes were calculated as previously described[41] and are shown as "density" maps in red. The calculated overlap volumes between antibody and FcεRIα or CD23 are shown in the tables. FcεRIα and CD23 are shown for clarity with a transparent surface rendering.

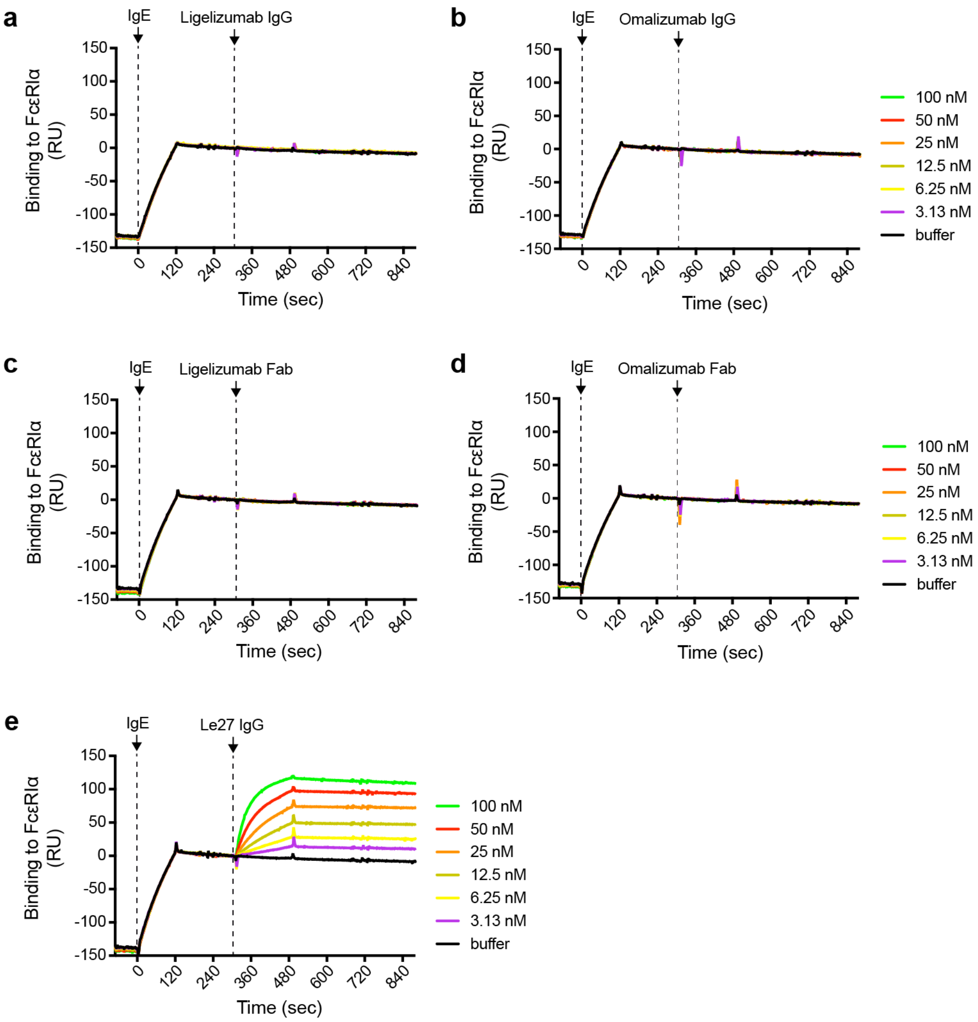

Supplementary Figure 5 | Interaction of ligelizumab or omalizumab IgG with FcεRI-bound IgE. Dose- and time-dependent binding of ligelizumab (**a**) and omalizumab IgG (**b**) and ligelizumab and omalizumab Fab fragments (**c**, **d**) to FcεRI-complexed human recombinant Sus11-IgE was measured by SPR. (**e**) The monoclonal anti-IgE antibody Le27, which recognizes and epitope in the Cε4 domain of IgE, was included as positive control. Each color in the sensorgram refers to an individual measurement cycle for the indicated of anti-IgE proteins. The black line refers to the baseline buffer control. Black arrows indicate the time of injection for IgE and anti-IgE proteins. Source data are provided as Source Data file.

Supplementary Table 1 | Binding kinetics of ligelizumab and omalizumab for IgE-$Fc_{3\text{-}4}$ variants.

Anti-IgE antibody	Target	Association k_a (M^{-1} s^{-1})	Dissociation k_d (s^{-1})	Affinity K_D (pM)
Omalizumab IgG	C328 IgE-$Fc_{3\text{-}4}$	4.5×10^{6}	2.9×10^{-3}	639.5
Ligelizumab IgG	C328 IgE-$Fc_{3\text{-}4}$	1.3×10^{7}	1.4×10^{-4}	11.0
Omalizumab IgG	C335 IgE-$Fc_{3\text{-}4}$	7.7×10^{6}	4.9×10^{-3}	608.2
Ligelizumab IgG	C335 IgE-$Fc_{3\text{-}4}$	1.1×10^{7}	6.5×10^{-3}	640.4

Supplementary Table 2 | Data collection and refinement statistics (molecular replacement)

	IgE-Fc:Ligelizumab-scFv
**Data collection**	
Space group	P212121 (19)
Cell dimensions	
a, *b*, *c* (Å)	75.1, 104.3, 124.8
α, β, γ (°)	90, 90, 90
Resolution (Å)	47.9 (3.65)*
R_{sym} or R_{merge}	33.1 (192)
$I / \sigma I$	8.4 (1.3)
Completeness (%)	100 (100)
Redundancy	10.9 (10.9)
**Refinement**	
Resolution (Å)	20-3.65
No. reflections	11142 (1096)
R_{work} / R_{free}	29.06/29.67
No. atoms	
Protein	6822
Ligand/ion	111
Water	0
B-factors	
Protein	113.0
Ligand/ion	124.1
Water	N/A
R.m.s. deviations	
Bond lengths (Å)	0.05
Bond angles (°)	0.94

Data collected from a single crystal at 100˚K.
*Values in parentheses are for highest-resolution shell
