# Supplementary material for: The mechanistic and functional profile of the therapeutic anti-IgE antibody ligelizumab differs from omalizumab
Source: Nat Commun. 2020 Jan 8;11:165. doi: 10.1038/s41467-019-13815-w (PMC6949303; doi:10.1038/s41467-019-13815-w)
Supplement: Supplementary file 3 — Reporting Summary [file 41467_2019_13815_MOESM3_ESM.pdf]

nature research

Corresponding author(s): Theodore S. Jardetzky
Alexander Eggel

Last updated by author(s): Nov 8, 2019

Reporting Summary

Nature Research wishes to improve the reproducibility of the work that we publish. This form provides structure for consistency and transparency in reporting. For further information on Nature Research policies, see Authors & Referees and the Editorial Policy Checklist.

Statistics

For all statistical analyses, confirm that the following items are present in the figure legend, table legend, main text, or Methods section.

n/a	Confirmed	
☐	☒	The exact sample size (*n*) for each experimental group/condition, given as a discrete number and unit of measurement
☐	☒	A statement on whether measurements were taken from distinct samples or whether the same sample was measured repeatedly
☐	☒	The statistical test(s) used AND whether they are one- or two-sided<br>*Only common tests should be described solely by name; describe more complex techniques in the Methods section.*
☒	☐	A description of all covariates tested
☐	☒	A description of any assumptions or corrections, such as tests of normality and adjustment for multiple comparisons
☐	☒	A full description of the statistical parameters including central tendency (e.g. means) or other basic estimates (e.g. regression coefficient) AND variation (e.g. standard deviation) or associated estimates of uncertainty (e.g. confidence intervals)
☐	☒	For null hypothesis testing, the test statistic (e.g. F, t, r) with confidence intervals, effect sizes, degrees of freedom and P value noted<br>*Give P values as exact values whenever suitable.*
☒	☐	For Bayesian analysis, information on the choice of priors and Markov chain Monte Carlo settings
☒	☐	For hierarchical and complex designs, identification of the appropriate level for tests and full reporting of outcomes
☒	☐	Estimates of effect sizes (e.g. Cohen's d, Pearson's r), indicating how they were calculated

Our web collection on statistics for biologists contains articles on many of the points above.

Software and code

Policy information about availability of computer code

Data collection	Data collection was performed using Microsoft Excel (Version 14-16) and GraphPad Prism (Version 5-7).
Data analysis	Data analysis was performed in GraphPad Software Prism 5-7.

For manuscripts utilizing custom algorithms or software that are central to the research but not yet described in published literature, software must be made available to editors/reviewers. We strongly encourage code deposition in a community repository (e.g. GitHub). See the Nature Research guidelines for submitting code & software for further information.

Data

Policy information about availability of data

All manuscripts must include a data availability statement. This statement should provide the following information, where applicable:

- Accession codes, unique identifiers, or web links for publicly available datasets
- A list of figures that have associated raw data
- A description of any restrictions on data availability

There are no restrictions on data availability. Raw data are provided in the Source Data file. All other data can be provided upon request.

Field-specific reporting

Please select the one below that is the best fit for your research. If you are not sure, read the appropriate sections before making your selection.

☒ Life sciences ☐ Behavioural & social sciences ☐ Ecological, evolutionary & environmental sciences

For a reference copy of the document with all sections, see nature.com/documents/nr-reporting-summary-flat.pdf

Life sciences study design

All studies must disclose on these points even when the disclosure is negative.

Sample size	No power calculations were performed. Sample size was determined based on previous experiments and expected significance levels.
Data exclusions	No data were excluded, unless a data point was clearly identified as outlier (3 standard deviations away from mean value).
Replication	Individual experiments were repeated at least twice with similar results, unless otherwise stated. Data is shown as representation of one experiment or as a combination of multiple independent experiments, as indicated in the text.
Randomization	Samples were chosen and assigned in an unbiased and randomized way.
Blinding	The investigators were not blinded during experimentation or data analysis.

Reporting for specific materials, systems and methods

We require information from authors about some types of materials, experimental systems and methods used in many studies. Here, indicate whether each material, system or method listed is relevant to your study. If you are not sure if a list item applies to your research, read the appropriate section before selecting a response.

Materials & experimental systems

n/a	Involved in the study	
☐	☒	Antibodies
☐	☒	Eukaryotic cell lines
☒	☐	Palaeontology
☐	☒	Animals and other organisms
☐	☒	Human research participants
☒	☐	Clinical data

Methods

n/a	Involved in the study	
☒	☐	ChIP-seq
☐	☒	Flow cytometry
☒	☐	MRI-based neuroimaging

Antibodies

Antibodies used	Ligelizumab as well as omalizumab antibodies and fragments were produced and kindly provided by Novartis Pharma AG (Basel, Switzerland). Sus11-IgE, JW8-IgE and the anaphylactogenic monoclonal anti-IgE antibody Le27 were purchased from NBS-C BioScience (Vienna, Austria). Monoclonal anti-human CD40 antibody was purchased (Enzo Life Sciences, NY, USA). The wild type C328 IgE-Fc3-4 and the mutated C335 IgE-Fc3-4 were produced in our laboratory as previously described. For flow cytometry we used the following antibodies: anti-human IgE FITC (clone Ige21, Thermo Fisher Scientific, MA, USA), monoclonal mouse anti-human FcεRIα APC (clone AER-37, Thermo Fisher Scientific, MA, USA) and the appropriate isotype controls monoclonal mouse IgG1,κ Isotype control FITC (Thermo Fisher Scientific, MA, USA) and mouse IgG2b Isotype control APC (Thermo Fisher Scientific, MA, USA), monoclonal rat anti-mouse CD200R FITC (clone OX-110, Bio-Rad, CA, USA), monoclonal rat anti-mouse CD117 PE (clone 2B8, Thermo Fisher Scientific, MA, USA), monoclonal mouse anti-human CD19 APC (clone HIB19, BD Bioscience), monoclonal mouse anti-human CD23 PE (clone EBVCS-5, Biolegend, CA, USA), monoclonal mouse anti-human CD1c PE-Cy7 (clone L161, Biolegend, CA, USA). For basophil activation testing the anti-human CCR3 and anti-human CD63 antibody staining mix from the Flow CAST® kit was used (Bühlmann Laboratories AG, Schönenbuch, CH). For ELISpot and IgE cell supernatant ELISA the human IgE ELISpotBASIC kit (Mabtech, Nacka Strand, Sweden) was used.
Validation	All antibodies used in this study were validated for flow cytometry, Western Blot, ELISA or ELISpot by the provider or manufacturer.

Eukaryotic cell lines

Policy information about cell lines

Cell line source(s)	RPMI 8866 has been established from the peripheral blood of a 51-year-old American woman with chronic myelogenous leukaemia in May 1966. The B lymphoid cell line expresses a relatively high amount of calcitonin receptors, membrane bound Ig and soluble IgE-binding factor (Fc epsilon RII/CD23).
Authentication	RPMI8866 cells were analyzed from CD19, CD20 and CD23 expression on the cell surface by flow cytometry.
Mycoplasma contamination	Cells were not tested for mycoplasma contamination.
Commonly misidentified lines (See ICLAC register)	Does not apply.

Animals and other organisms

Policy information about studies involving animals; ARRIVE guidelines recommended for reporting animal research

Laboratory animals	Mice transgenic for human FcεRIα (B6.Cg-Fcer1atm1Knt Tg(FCER1A)1Bhk/J) on a mixed C57BL/6 J - C57BL/6 N background were obtained from Prof J.-P. Kinet.
Wild animals	Does not apply.
Field-collected samples	Does not apply.
Ethics oversight	All animal experimentation was approved by the Bernese cantonal ethics committee (authorization BE66/18).

Note that full information on the approval of the study protocol must also be provided in the manuscript.

Human research participants

Policy information about studies involving human research participants

Population characteristics	Whole blood donations from healthy and allergic volunteers were collected.
Recruitment	Patients were randomly recruited through public advertisement at the University Hospital Bern.
Ethics oversight	The study was approved by the swiss ethics committee (KEK 2018-00204).

Note that full information on the approval of the study protocol must also be provided in the manuscript.

Flow Cytometry

Plots

Confirm that:

☒ The axis labels state the marker and fluorochrome used (e.g. CD4-FITC).

☒ The axis scales are clearly visible. Include numbers along axes only for bottom left plot of group (a 'group' is an analysis of identical markers).

☒ All plots are contour plots with outliers or pseudocolor plots.

☒ A numerical value for number of cells or percentage (with statistics) is provided.

Methodology

Sample preparation	Primary human basophils and BDCA1+ dendritic cells (DCs) were isolated from whole blood donations. Human peripheral whole blood was obtained from volunteering donors, who provided informed consent in accordance with the Helsinki Declaration. The study was approved by the local ethics committee (KEK 2018-00204). Basophils and BDCA1+ DCs were enriched by Percoll density centrifugation of dextran-sedimented supernatants. Furthermore, basophils were purified with negative selection using the Milteny basophil isolation kit II (Miltenyi Biotec, Bergisch Gladbach, Germany). BDCA1+ DCs were isolated with positive selection using the Milteny human CD1c (BDCA-1+ dendritic cell isolation kit (Miltenyi Biotec, Bergisch). Cells were analyzed for purity via flow cytometry.<br><br>Peritoneal mast cells were identified in peritoneal lavages from euthanized mice, as CD45+, c-kit+, CD200R3+ cells.<br><br>RPMI8866 were cultured in RPMI+/+ medium at a density of 2.5 x 10^5 cells/ml in a 250ml cell culture flask (Greiner Bio One, Kremsmünster, AUT). One day before the experiment the cells were split 1:2 in RPMI+/+ medium.
Instrument	Flow cytometry was performed using a BD FACSCanto device (BD Bioscience, Franklin Lakes, NJ, USA)
Software	Results were evaluated with FlowJo Version 10.1 (Ashland, OR, USA)and BD FACSDIVA™ Software.
Cell population abundance	- purified primary human basophils: > 90%<br>- purified primary human BDCA1+ DCs: > 85%<br>- cultured RPMI8866 cells: ~100%<br>- peritoneal mouse mast cells: <1%
Gating strategy	- purified primary human basophils: CD123+, CD193+ cells<br>- purified primary human BDCA1+ DCs: SSC-low, CD1c+ cells<br>- cultured RPMI8866 cells: SSC-high, CD19+, CD23+ cells<br>- peritoneal mouse mast cells: CD45+, c-kit+, CD200R3+ cells

☒ Tick this box to confirm that a figure exemplifying the gating strategy is provided in the Supplementary Information.
